# Ocular fundus and optical coherence tomography (OCT) findings in children prenatally exposed to opioid maintenance therapy (OMT)

**DOI:** 10.1186/s12886-025-04342-y

**Published:** 2025-11-13

**Authors:** Olav H. Haugen, John-Thomas Michelet, Anne Kathinka Aslaksen, Gro Horgen, Jon Skranes

**Affiliations:** 1 Department of Ophthalmology, Haukeland University Hospital, Bergen, Norway; 2 Department of Clinical Medicine K1, Faculty of Medicine, University of Bergen, Bergen, Norway; 3 Department of Ophthalmology, Sørlandet Hospital, Arendal, Norway; 4 Department of Paediatrics, Sørlandet Hospital, Arendal, Kristiansand, Norway; 5 Department of Optometry, Radiography and Lighting Design, University of South- Eastern Norway, Kongsberg, Norway

**Keywords:** Optical coherence tomography, Opioid maintenance therapy, Neurodevelopment

## Abstract

**Background:**

Optical coherence tomography (OCT) has been of great importance in the diagnostics and follow-up of many retinal diseases the last 25 years. More recently, this technology has also proved useful in the evaluation and follow-up of various neurological and psychiatric disorders. Also, certain prenatal impacts, such as prematurity and foetal alcohol spectrum disorder have shown abnormal findings on OCT. The aim of this study was to examine children that have been prenatally exposed to opioid maintenance therapy (OMT) with OCT.

**Methods:**

As a part of a comprehensive study on visual and visual-motor function, 63 children aged 5–13 years exposed prenatally to OMT and 77 age-matched controls were examined with OCT, collecting data from the peripapillary retinal nerve fibre layer (RNLF) and the macular region. The participants were recruited at two different centres with different OCT-instruments.

**Results:**

A highly significant thinning of the peripapillary RNFL was found in the OMT-exposed children compared to the controls at both study centres. The macular data did also show a reduced retinal thickness and volumes in several macular regions in the OMT-exposed children, but to a variable degree at the two participating study centres. The reasons for this variability remain uncertain. There was no correlation between the OCT data and visual acuity.

**Conclusions:**

This is the first study reporting OCT-data from children prenatally exposed to OMT. The significant thinning of the peripapillary RNFL in the OMT-exposed children most probably reflects a negative impact of the opioid substances on the foetal neurodevelopment. Further OCT-studies on OMT-exposed children should be carried out.

**Supplementary Information:**

The online version contains supplementary material available at 10.1186/s12886-025-04342-y.

## Background

From the introduction of optical coherence tomography (OCT) in clinical ophthalmology in the early 2000 s, this technology has revolutionized diagnostics in retinal diseases [1]. Since then, there has been a gradual improvement regarding image resolution, making it possible to visualize the anatomical structure of the retina down to impressive details. First and foremost, OCT has literally led to a paradigm change concerning the treatment of macular conditions such as age-related macular degeneration (AMD), diabetic macular oedema, and macular holes [2]. However, OCT has also demonstrated that the structure of the macular area (thickness and volume) as well as the optic nerve head shows characteristic changes associated with other conditions like the refractive state of the eye [3] and prematurity [4, 5]. Recently, changes of the retinal structure on OCT have also been demonstrated in different neurological conditions such as multiple sclerosis [6] and Parkinson’s disease [7], as well as psychiatric disorders [8], although uncertainties exist concerning the relationship between these changes and the clinical course of the diseases. Taken together, these findings reflect the fact that the retina anatomically and developmentally is an extension of the brain.

From studies on children born prematurely, thinning of the peripapillary retinal nerve fibre layer (RNFL) on OCT has been found in very preterm (gestational age < 32 weeks), either related to the presence of severe of ROP [5] or not [9]. Even more pronounced RNFL thinning has been shown in extremely preterm born children (≤ 28 weeks), independent of ROP [10]. We have not found studies demonstrating RNFL thinning in preterm born children with gestational age between 32 and 37 weeks. In addition, OCT-studies on macular anatomy in preterm children have shown increased central macular thickness compared to full-term born children [11, 12]. Jacobson et al. [13] have also shown that the ganglion cell layer on retinal OCT demonstrated focal thinning in individuals with spastic cerebral palsy.

Concerning the impact of other prenatal factors on the early development of the retinal structure, this has mostly been studies on maternal smoking or the use of alcohol. In a study from 2011, Pueyo et al. [14] showed that maternal smoking during pregnancy caused a reduction of mean RNFL thickness, although no difference could be demonstrated in visual acuity, colour vision or stereoacuity. The effect of RNFL thinning from prenatal exposure to cigarette smoking has been verified also by others [15]. Menezes et al. [16] found significant thinning of the peripapillary RNFL in young adults with foetal alcohol spectrum disorder (FASD) compared to healthy controls, a finding recently confirmed by Lehikoinen et al. [17] and Gyllencreutz et al. [18].

A literature search regarding OCT-findings with the prenatal exposure of other possible harmful substances or drugs (e.g. anti-depressant, antipsychotics, benzodiazepines, amphetamine and methamphetamine, cocaine and cannabis) gave no result.

We have studied a cohort of children who were exposed to maternal opioid maintenance therapy (OMT) during pregnancy. In this cohort, we have recently reported a higher prevalence of strabismus and nystagmus, as well as a reduced visual acuity and a lower accommodation amplitude compared to normal controls [19]. In a second publication with the same patient material, we demonstrated a higher frequency of motor problems, as well as problems with visual-motor integration, in the OMT-exposed children [20]. Furthermore, we have also recently documented reduced volumes of numerous brain structures on MRI images in OMT-exposed children compared to the control group [21].

The aim of the present paper was to study the posterior segment of the eye in the same patient sample. The neuronal endogenous opioid system of the fetus plays an important role in modulating several functions in the developing brain, such as cell growth, differentiation, maturation, and myelin formation. It is known that exogenous opioid substances influence these processes [22]. With this in mind, and knowing the impact of other conditions like prematurity and FASD reported in previous OCT-studies, our hypothesis was that prenatal exposure to OMT also may cause changes on OCT. In particular, we wanted to examine the structure of the optic nerve head and macular region using OCT. To our knowledge, OCT-data has not previously been reported in prenatally OMT-exposed children.

## Methods

### Patients and control groups

This study was an integrated part of a more extensive observational cohort study during the years 2018–2021, comprising visual and motor functions in children 5–13 years of age prenatally exposed to OMT (*n* = 63), and an age-matched control group (*n* = 63). The study was a collaboration between two different hospitals in Norway, Sørlandet Hospital (centre A) and Haukeland University Hospital (centre B), and recruitment of children to the study took place at both centres. Inclusion criteria for the OMT-group were: mother had been on OMT during pregnancy, and age at examination was between 5 and 13 years. The response rate was high at both study centres, in particular at centre A (95% in the OMT-group and 79% in the control group), but also at centre B (74% in the OMT-group and 50% in the control group). The details of the recruitment procedure have been described in a previous publication [19].

During the recruitment period, similar numbers of OMT-exposed children were entered into the study at the two participating centres (*n* = 32 at centre A and *n* = 31 at centre B). However, there were significantly more control children recruited at centre A (*n* = 53) than at centre B (*n* = 10). As the OCT instruments were different at the two centres, comparison of the OCT data between the OMT-exposed children and the controls had to be done separately, making it necessary to include more control children at centre B after inclusion in the main study had been closed, exclusively for the OCT-study. Thus, 14 additional healthy controls were recruited, making up a control group at centre B of totally 24 children. All children in the study except ten were born at term. Among the ten children born preterm (gestational age < 37 weeks), five were in the OMT-group (mean gestational age 35.0 ± 0.7 weeks) and five were in the control group (35.2 ± 0.8 weeks). No children had a gestational age less than 34 weeks.

### Ocular fundus and OCT examination

As part of the study protocol, all the participating children (both the OMT-exposed and the control group) underwent a clinical examination of the ocular fundus after the instillation of dilating eye drops in both eyes (cyclopentolate 1%). This was done either with indirect ophthalmoscopy and a 20D lens, or with the slit lamp and a 90D lens. In addition, a colour fundus photo was taken, primarily for a closer evaluation of the optic disc. At centre A, the colour fundus photo was obtained by the OCT instrument (Topcon DRI OCT Triton, Topcon Corp., Tokyo, Japan), whereas centre B used a Zeiss fundus camera (Zeiss FF450 plus IR, Carl Zeiss Meditec, Jena, Germany) for this purpose. Both instruments have programs with a calliper function for the measurements of retinal structures on the images, and horizontal and vertical optic disc diameter for each eye was recorded (mean values of three measurements).

**Fig. 1:**
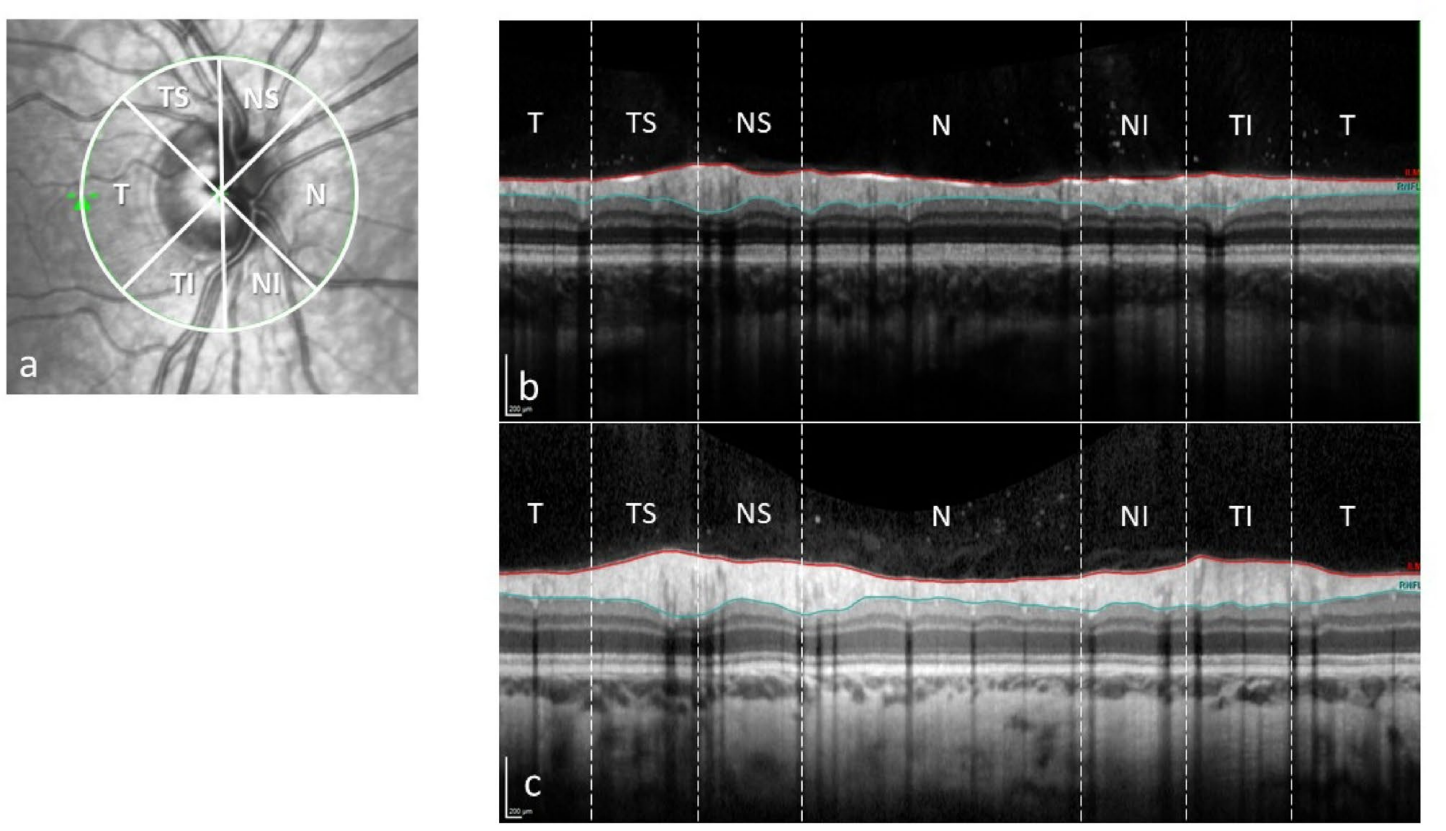
Peripapillary retinal nerve fibre layer; sectors (**a**), thickness scan (between the red and blue lines) from an OMT-patient (**b**) and a normal control (**c**). T: temporal, TS: temporal superior, NS: nasal superior, N: nasal, NI: nasal inferior, TI: temporal inferior

Furthermore, an OCT of the optic nerve head (peripapillary RNFL) and macula (retinal thickness and volumes) was done on both eyes. For the OCT examinations, there were also different instruments at the two centres; Topcon DRI OCT Triton (same instrument as above) at centre A, and Heidelberg Spectralis (Heidelberg Engineering GmbH, Heidelberg, Germany) at centre B. Mean average peripapillary RNFL thickness of the nerve head and of the six different sectors (temporal, nasal, nasal superior, nasal inferior, temporal superior and temporal inferior) was measured at both centres. Peripapillary RNFL is measured with a circle scan (diameter 3.0 mm) around the optic nerve. On an out-stretched scan of this circle, the inner limiting membrane and the border between the RNFL and the ganglion cell layer are outlined. From these two lines, the RNFL thickness is measured (Fig. 1).

**Fig. 2:**
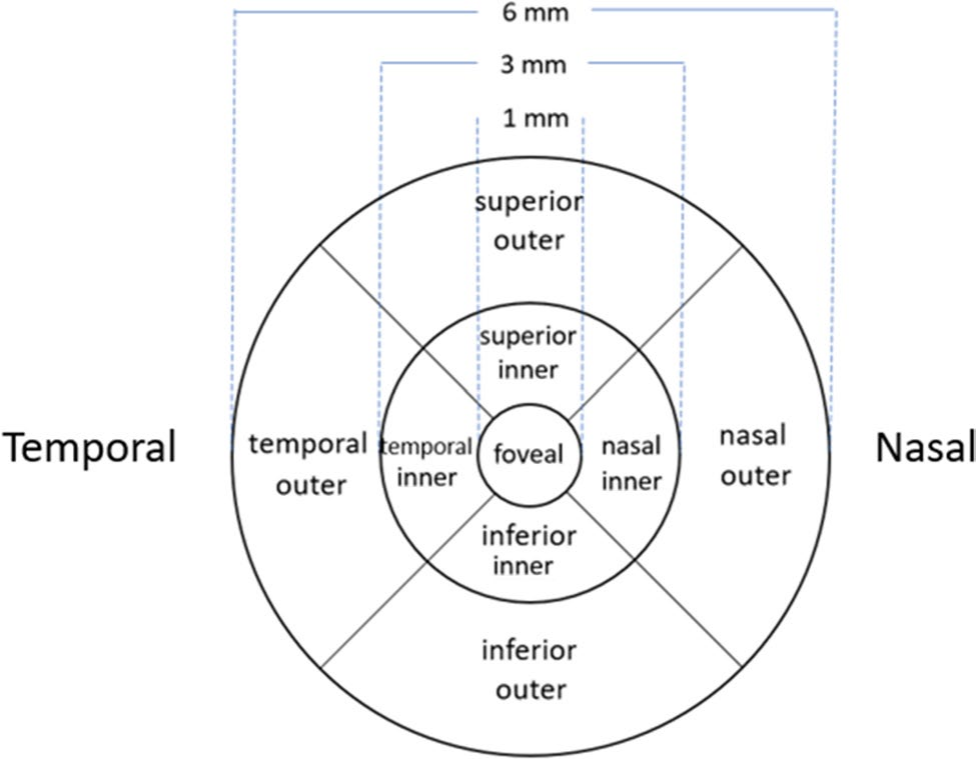
The ETDRS macular grid with nine subfields

In macular OCT measurements, mean thickness of the nine subfields defined in the Early Treatment Diabetic Retinopathy Study (ETDRS) grid [23] was measured. Centrally, this grid has an inner circle around the fovea, with a diameter of 1 mm. Two additional, concentric circles outside the first circle, with diameters of 3 and 6 mm, respectively, define an inner zone (parafoveal, between the first and second circle) and an outer zone (perifoveal, between the second and third circle). Furthermore, the inner and outer zones are divided into 4 subfields by oblique lines, creating upper, lower, nasal and temporal sectors in both the inner and outer zones (Fig. 2).

Mean volume of each of the nine subfields was only possible to measure with the Heidelberg OCT instrument at centre B, while total macular volume (the whole grid area) could be measured at both centres. At centre A, an experienced ophthalmologist (JTM) did both the OCT examinations and the OCT image analyses, while at centre B, the examinations were done by an experienced ophthalmic photographer, and the image analyses were performed by an experienced ophthalmologist (OHH). The OCT scans were checked for centration and segmentation errors. When transferring the data from the OCT-instruments to the statistic program, all the data were also manually checked for outliers and transfer errors.

In order to explore any relation between the OCT-data and brain volume data from the MRI-article [21], we did correlation analyses between the average RNLF measurement of the optic nerve head and the 34 different MRI-measured volumes, performing separate analyses for left and right sides (left eye data to left brain volumes and vice versa).

### Statistical analyses

SPSS version 29 (IBM Corp., Armonk, NY, USA), was used in the analyses. To compare OCT measurements between the OMT-exposed and the control group, as well as within the OMT-group, Student’s t-test was used, assuming a normal distribution of the means. Correlation analyses were performed using Pearson’s correlation coefficient. A two-sided *p*-value of ≤ 0.05 was considered as level of significance.

## Results

The distribution of sex and age for the OMT-exposed children and the controls groups at the two study centres is presented in Table 1.

**Table 1:** Age-, sex and refractive distribution (right eyes) among the OMT-exposed children and the control group at the two study centres

	OMT-exposed group	Control group	*p*-value
**Centre A**			
Number of participants	32	52	
Sex (male/female)	16/16	23/30	0.55
Age at examination, months (mean ± SD)	94 0.4 ± 26.0	103.9 ± 26.7	0.12
Spherical equivalent, D (mean ± SD)	+ 1.69 ± 2.20	+ 1.10 ± 1.15	0.13
**Centre B**			
Number of participants	31	24	
Sex (male/female)	15/16	17/7	0.09
Age at examination, months (mean ± SD)	103.4 ± 27.5	112.1 ± 26.6	0.24
Spherical equivalent, D (mean ± SD)	+ 1.25 ± 1.59	+ 0.53 ± 0.44*	0.19

*At Centre B, 14 additional children were recruited to the control group especially for the OCT examinations (see Method chapter). Refraction was not measured in these children, and the refractive value for the control group at centre B in the table is thus only based on the ten control children from the main study

**Fig. 3:**
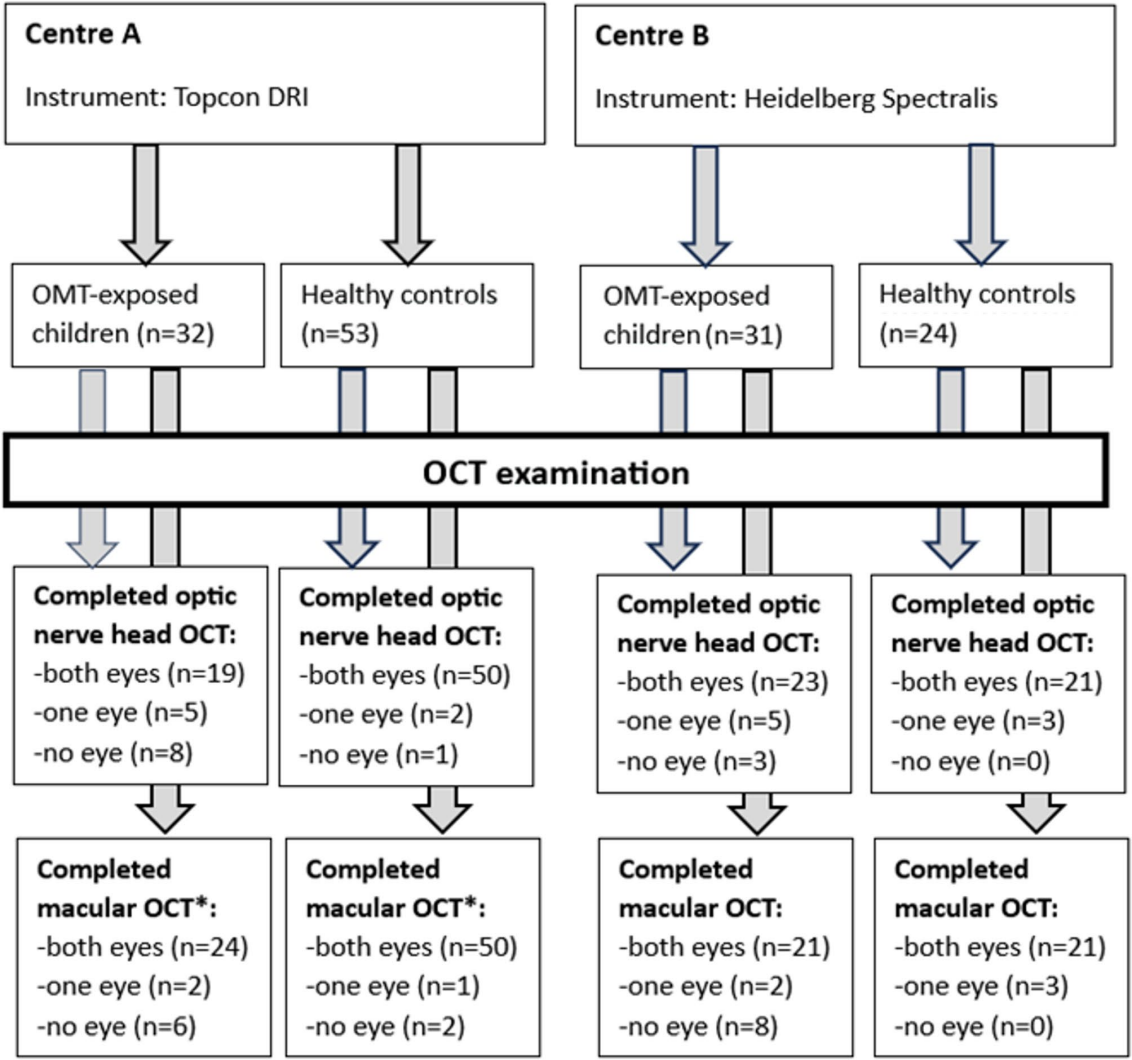
Overview of prenatally OMT-exposed children and healthy control group examined with macular and optic nerve head OCT. *The Topcon instrument does not calculate the volume of the nine ETDRS subfields, thus only total macular volume could be measured

Not all children managed to cooperate sufficiently for a complete OCT-examination. For a few children this was due to a general insufficient ability to cooperate at the OCT-instrument, while others had a poor fixation during parts of the examination, making it necessary to exclude one or both eyes. Figure 3 displays in detail the number of children at the two participating centres who completed the different OCT-examinations.

In addition, important background information of the OMT-exposed children at the two study centres is presented in Table 2.

**Table 2:** Clinical characteristics for the OMT-exposed children at centre A and B

Variable	Centre A	Centre B	*p*-value
Age at examination, months (mean ± SD)	94.4 ± 26.0	103.4 ± 27.5	0.18
Sex, m/f	16/16	15/16	
Gestational age, months (mean ± SD)	39.0 ± 1.9	39.5 ± 1.6	0.28
Birth weight, g (mean ± SD)	3056 ± 606	3288 ± 487	0.10
Head circumference at birth, cm (mean ± SD)	33.8 ± 1.8	34.6 ± 1.7	0.12
Opioid substance exposure, n (%)			0.04
Methadone	14 (44)	6 (19)	
- Daily dosage, mg (mean ± SD)	97.4 ± 43.1	150.0 ± 123.6	0.17
Buprenorphine	18 (56)	25 (81)	
- Daily dosage, mg (mean ± SD)	10.8 ± 8.4	17.2 ± 6.7	0.02
Side abuse, n (%)	8 (24)	19 (61)	0.004
Spherical equivalent right eye, D (mean ± SD)	+ 1.69 ± 2.20	+ 1.25 ± 1.59	0.41
Visual acuity best eye, logMAR (mean ± SD)	0.06 ± 0.14	0.04 ± 0.09	0.40
Nystagmus, n (%)	6 (19)	4 (13)	0.53

### Fundus examination

The clinical fundus examination did not reveal congenital malformations or other structural retinal pathology in any of the OMT-exposed or control children. Additionally, there were no significant differences in the optic disc diameter measurements on the fundus photos in the OMT-exposed children compared to the control group (Supplemental material, Table 1).

### OCT examination

Table 3 shows the mean thickness of the peripapillary RNFL, both in average and for the six different peripapillary sectors.

**Table 3:** Peripapillary retinal nerve fibre layer (RNFL) thickness on OCT in the OMT-exposed and the control group; data for centre A and B presented separately

Retinal nerve fiber layer thickness (µ), mean ± SD, eye and optic nerve head sector	OMT-exposed group	Control group	*p*-value	Cohen’s d
**Centre A**				
*Right eye*				
Average Temporal Temporal superior Nasal superior Nasal Nasal inferior Temporal inferior	96.8 ± 12.5 72.8 ± 8.1 137.6 ± 21.1 108.1 ± 21.0 78.7 ± 16.6 108.8 ± 32.3 135.6 ± 25.0	107.1 ± 9.5 74.4 ± 9.8 146.9 ± 17.7 118.7 ± 27.8 92.4 ± 13.6 128.0 ± 26.3 149.4 ± 22.3	< 0.001 0.49 0.056 0.12 < 0.001 0.01 0.022	0.98 0.18 0.50 0.41 0.94 0.68 0.60
*Left eye*				
Average Temporal Temporal superior Nasal superior Nasal Nasal inferior Temporal inferior	93.5 ± 11.8 68.0 ± 5.9 130.4 ± 15.4 114.1 ± 23.3 75.7 ± 18.2 103.0 ± 34.0 130.3 ± 16.5	106.0 ± 9.7 72.3 ± 8.4 143.5 ± 19.4 123.9 ± 25.2 89.6 ± 13.7 128.2 ± 23.5 146.4 ± 21.6	< 0.001 0.035 0.008 0.13 < 0.001 < 0.001 0.003	1.22 0.56 0.71 0.40 0.92 0.94 0.80
**Centre B**				
*Right eye*				
Average Temporal Temporal superior Nasal superior Nasal Nasal inferior Temporal inferior	87.2 ± 9.4 67.3 ± 12.2 122.9 ± 18.4 94.0 ± 18.8 67.6 ± 14.6 91.3 ± 27.6 119.3 ± 22.2	103.2 ± 6.0 76.4 ± 10.5 146.9 ± 19.8 113.7 ± 21.8 73.8 ± 12.2 117.1 ± 17.3 149.9 ± 16.3	< 0.001 0.006 < 0.001 0.001 0.11 < 0.001 < 0.001	1.98 0.80 1.26 0.98 0.46 1.10 1.55
*Left eye*				
Average Temporal Temporal superior Nasal superior Nasal Nasal inferior Temporal inferior	86.2 ± 9.8 65.4 ± 10.9 120.8 ± 24.4 103.0 ± 21.1 61.9 ± 17.5 90.5 ± 23.7 122.4 ± 19.0	102.8 ± 6.6 73.4 ± 9.0 145.7 ± 12.1 127.0 ± 16.8 72.5 ± 12.2 116.5 ± 20.5 145.9 ± 15.5	< 0.001 0.009 < 0.001 < 0.001 0.021 < 0.001 < 0.001	2.00 0.80 1.29 1.26 0.70 1.17 1.36

OCT: optical coherence tomography, OMT: opioid maintenance therapy

**Fig. 4:**
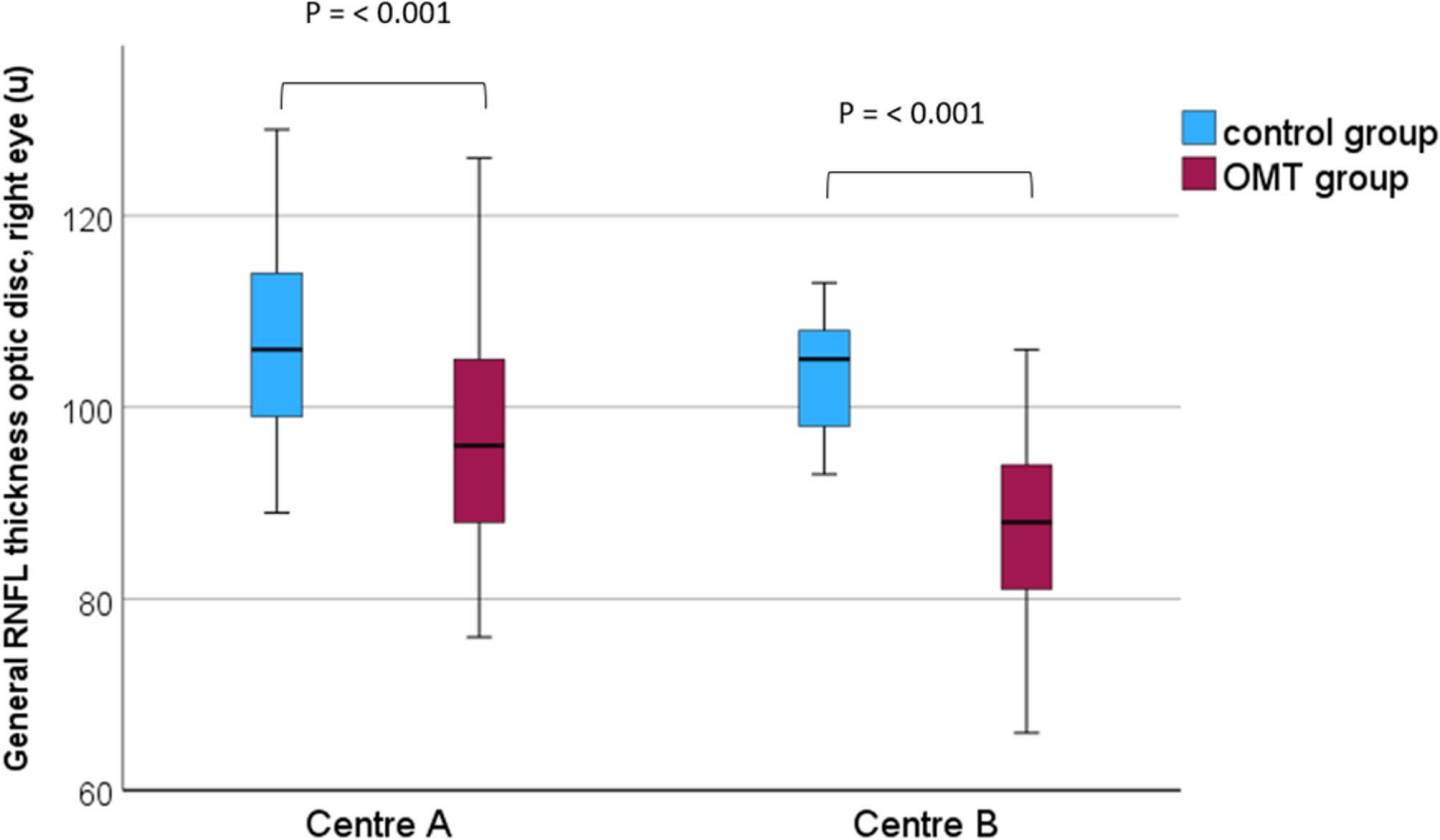
General retinal nerve fibre layer thickness of the optic disc (µ), right eye; centre **A** and **B**

As shown in the table, a highly significant thinning of the peripapillary RNFL in the OMT-exposed group, both for the average peripapillary area (Fig. 4) and for most of the peripapillary sectors, was found at both centres, compared to the control group.

The measurements of macular thickness differed significantly between the two study centres. At centre A, only two of the outer subfields (outer superior in the right eyes and outer inferior in the left eyes) showed significant differences in thickness between the OMT-group and the control group, macular thickness being reduced in the OMT-group. This was in contrast to the measurements at centre B, where a highly significant thinning of the retina was found in all subfields, except the central, in the OMT-group (Table 4).

**Table 4:** Macular thickness measurements on OCT in the OMT-exposed and the control group; data for centre A and B presented separately

Retinal thickness (µm), mean ± SD, eye and macular subfield	OMT-exposed group	Control group	*p*-value	Cohen’s d
**Centre A**				
*Right eye*				
Central Superior, inner Temporal, inner Inferior, inner Nasal, inner Superior, outer Temporal, outer Inferior, outer Nasal, outer	238.8 ± 19.8 315.1 ± 16.9 304.4 ± 16.2 314.8 ± 15.9 315.3 ± 17.2 275.7 ± 16.7 264.0 ± 16.2 268.4 ± 19.1 291.6 ± 21.2	239.5 ± 19.7 317.8 ± 12.9 306.1 ± 12.6 316.0 ± 12.4 317.3 ± 14.1 283.0 ± 12.2 268.7 ± 12.2 274.3 ± 13.7 297.4 ± 13.9	0.89 0.46 0.62 0.74 0.60 0.04 0.16 0.13 0.15	0.03 0.18 0.12 0.08 0.13 0.54 0.35 0.38 0.35
*Left eye*				
Central Superior, inner Temporal, inner Inferior, inner Nasal, inner Superior, outer Temporal, outer Inferior, outer Nasal, outer	240.3 ± 18.7 316.3 ± 20.8 304.3 ± 15.6 313.7 ± 15.6 317.2 ± 16.7 279.4 ± 15.2 263.3 ± 17.4 262.2 ± 22.6 292.9 ± 20.0	240.6 ± 18.7 318.5 ± 15.8 304.6 ± 16.8 314.5 ± 17.2 317.1 ± 16.7 281.7 ± 16.3 266.9 ± 14.7 272.1 ± 13.9 297.0 ± 16.4	0.95 0.58 0.94 0.85 0.99 0.57 0.35 0.02 0.35	0.02 0.14 0.02 0.05 −0.003 0.15 0.23 0.57 0.23
**Centre B**				
*Right eye*				
Central Superior, inner Temporal, inner Inferior, inner Nasal, inner Superior, outer Temporal, outer Inferior, outer Nasal, outer	269.1 ± 24.8 339.7 ± 12.2 328.8 ± 12.1 337.3 ± 12.8 342.6 ± 13.3 296.7 ± 16.2 283.5 ± 14.0 284.4 ± 16.7 312.6 ± 16.4	271.6 ± 15.6 352.9 ± 11.0 338.7 ± 12.3 347.9 ± 12.5 350.6 ± 12.1 313.0 ± 9.4 300.0 ± 9.6 301.2 ± 7.7 327.4 ± 9.7	0.70 < 0.001 0.009 0.007 0.04 < 0.001 < 0.001 < 0.001 < 0.001	0.12 1.13 0.81 0.84 0.64 1.22 1.37 1.28 1.10
*Left eye*				
Central Superior, inner Temporal, inner Inferior, inner Nasal, inner Superior, outer Temporal, outer Inferior, outer Nasal, outer	268.2 ± 24.3 338.0 ± 10.2 328.1 ± 11.2 337.8 ± 12.6 342.0 ± 11.3 298.0 ± 20.2 284.2 ± 16.0 283.6 ± 13.9 311.8 ± 15.8	273.0 ± 15.8 352.7 ± 12.6 338.5 ± 12.5 349.5 ± 10.9 350.7 ± 13.6 312.3 ± 10.3 298.9 ± 12.1 299.7 ± 9.5 327.6 ± 12.1	0.44 < 0.001 0.006 0.002 0.03 0.005 0.001 < 0.001 < 0.001	0.23 1.28 0.88 0.99 0.69 0.90 1.05 1.36 1.13

OCT: optical coherence tomography, OMT: opioid maintenance therapy

**Fig. 5:**
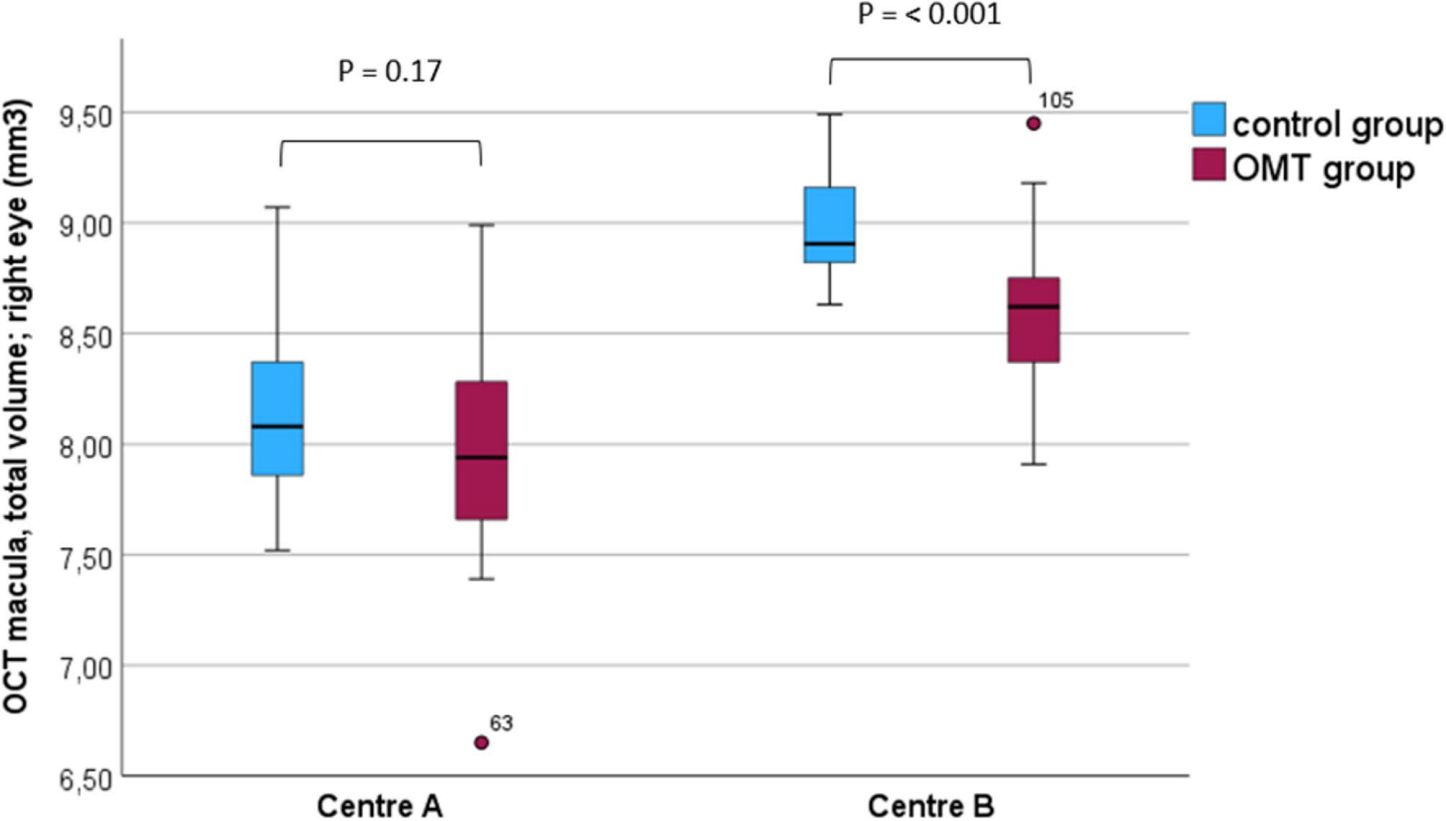
Total macular volume, right eye; centre **A** and **B**

Total volume of the macula was reduced in the OMT-group compared to the controls at both centres, but only with a significant difference at centre B (Fig. 5).

Volume measurements of the individual macular subfields could only be done at centre B, showing significantly reduced volume in the OMT-group in all subfields except for the central field (Table 5).

**Table 5:** Tissue volume measurements in the nine subfields of macula on OCT in the OMT-exposed and the control group; data from centre B

Retinal volume (mm^3^), mean ± SD, eye and macular subfield	OMT-exposed group	Control group	*p*-value	Cohen’s d
**Centre B**				
*Right eye*				
Central Superior, inner Temporal, inner Inferior, inner Nasal, inner Superior, outer Temporal, outer Inferior, outer Nasal, outer	0.21 ± 0.02 0.53 ± 0.02 0.52 ± 0.02 0.53 ± 0.02 0.54 ± 0.02 1.57 ± 0.09 1.50 ± 0.08 1.51 ± 0.09 1.66 ± 0.09	0.21 ± 0.01 0.56 ± 0.02 0.53 ± 0.02 0.55 ± 0.02 0.55 ± 0.02 1.66 ± 0.05 1.59 ± 0.05 2.00 ± 0.04 1.74 ± 0.05	0.77 < 0.001 0.007 0.004 0.05 < 0.001 < 0.001 < 0.001 < 0.001	0.09 1.13 0.85 0.90 0.60 1.23 1.36 1.30 1.11
*Left eye*				
Central Superior, inner Temporal, inner Inferior, inner Nasal, inner Superior, outer Temporal, outer Inferior, outer Nasal, outer	0.21 ± 0.02 0.53 ± 0.02 0.51 ± 0.02 0.53 ± 0.02 0.54 ± 0.02 1.58 ± 0.10 1.51 ± 0.09 1.50 ± 0.08 1.65 ± 0.08	0.22 ± 0.01 0.56 ± 0.02 0.53 ± 0.02 0.55 ± 0.02 0.55 ± 0.02 1.66 ± 0.06 1.58 ± 0.07 1.59 ± 0.05 1.74 ± 0.06	0.37 < 0.001 0.003 0.002 0.03 0.004 0.002 < 0.001 < 0.001	0.27 1.34 0.96 1.00 0.70 0.93 1.01 1.34 1.15

OCT: optical coherence tomography, OMT: opioid maintenance therapy

Among the OMT-exposed children with nystagmus (*n* = 10), one of six at centre A and all four at centre B managed to perform a complete OCT examination of the peripapillary RNFL. A thinning of the RNFL was found in all these children. However, a statistical comparison was only possible to carry out on the data from centre B (only one single child with nystagmus at centre A), showing a significant reduction in average mean RNFL thickness of 72.4 ± 5.9 µ in the nystagmus subgroup compared to 89.5 ± 7.6 µ in the non-nystagmus group (*p* < 0.001).

Concerning the macular OCT-examination of the children with nystagmus, three of the six at centre A and all four children at centre B were able to perform a complete macular OCT-examination. At centre A, there were three children with manifest nystagmus in all positions of gaze, whereas three only had gaze-induced nystagmus. At centre B, all four children with nystagmus had gaze-induced nystagmus. We have analysed the central subfield, finding an increased mean central retinal thickness at both centres, but only statistically significant at centre A (*p* = 0.02) and not in centre B (*p* = 0.53). The number of children with nystagmus at each centre were very small, and the results, both for the RNFL and the macular measurements should be interpreted with caution.

No correlation was found between visual acuity and peripapillary RNFL, or between visual acuity and macular thickness in the OMT-group. At both study centres, correlation analyses in the OMT-group between the total peripapillary RNFL data on OCT and multiple brain volumes revealed a significant correlation between average peripapillary RNFL and volumes of the rostral middle frontal gyrus both for the right and left side (centre A: *r* = 0.55 and *p* = 0.009, and *r* = 0.51 and p 0 0.03 respectively; centre B: *r* = 0.53 and *p* = 0.007, and *r* = 0.43 and *p* = 0.05, respectively). No such correlation was found in the control groups.

## Discussion

This study revealed a highly significant thinning of the peripapillary RFNL in the prenatally OMT-exposed children compared to the control group at both participating centres. This thinning was present both for the average measurement of the whole peripapillary area (Fig. 4) and for most of the individual sector areas of the optic nerve head (Table 3). The optic nerve and the neuroretina are developmentally both parts of the central nervous system, and a thinning of the peripapillary RNFL in the OMT-exposed children conceivably reflects a general disturbance in the foetal neurodevelopment. Other OCT-studies showing similar thinning in conditions such as extreme prematurity and foetal alcohol spectrum disorder (FASD) [9–12; 16–18] seem to support this view. However, as for other visually related conditions such as strabismus [19], it is important to point out that a possible confounder to this RNFL thinning is maternal smoking during pregnancy. Nearly all the mothers (95%) of the OMT-exposed children smoked regularly during the pregnancy, whereas only 3% of the mothers in the control group smoked. As pointed out in the introduction, previous OCT-studies on children have shown thinning of the RNFL in cases with maternal cigarette smoking during pregnancy [14, 15].

The OCT-measurements from the macular region differed significantly between the two study centres, making the macular data more difficult to interpret. At centre A, macular thickness data from only one of the outer subfields in the right and left eyes (superior outer and inferior outer, respectively) showed a significant thinning in the OMT-exposed group. However, in the right eye data, also the other three outer subfields (temporal outer, inferior outer, and nasal outer) were substantially thinner in the OMT-group, although not statistically significant. Data from centre B showed a significantly reduced macular thickness in all subfields except the central (Table 4). The total volume measurements showed reduced values in the OMT-exposed group at both centres, but only statistically significant at centre B (Fig. 5). The reason for these differences is not obvious. It is well known that measurements from different OCT instruments cannot be readily compared [24], as the different instruments define the borders of the retinal layers slightly differently. However, these differences do not explain the presence of significant group differences within one centre and not in the other. In order to explore the diverse results of the macular OCT measurements at the two study centres, the raw data for each single examination were also re-checked manually. This revealed two papillary and two macular OCT-examinations that were of technically low quality, and these were taken out of further analyses. However, this caused only minor changes on the results. In Table 2, important background information about the OMT-exposed children examined at the two different centres is presented. This shows that a significantly higher percentage of the children at centre A compared to centre B had been exposed to methadone, and, in addition, both substances had been given at higher doses at centre B compared to centre (A) Moreover, side abuse during pregnancy had been much more frequently recorded among the mothers of the OMT-exposed children at centre (B) One may speculate that the impact of prenatal OMT-exposure on the retinal structure of macula could be dose-dependent, eventually also related to side-abuse, or due to combined effects of multiple factors that differed between the two study centres. Further research is needed to explore this matter.

It is interesting that recent OCT-studies have documented that drug abuse in adult life also may cause alterations, both in RNFL and macular thickness. However, these studies have shown contradictory results. Two studies on young adults with multiple substance abuse have shown increased RNFL thickness and decreased macular thickness [25, 26], whereas a study on older cocaine users has revealed a significant RNFL thinning with no difference in macular thickness [27]. Furthermore, a prospective study on subjects with chronic opioid use treated four weeks with buprenorphine/naloxone maintenance therapy showed a significant thinning of the RNFL at the end of the treatment period [28]. As the optic nerve and retina are parts of the central nervous system and given the fact that RNFL thickness reflects the number of nerve fibres from retina passing to the optic nerve, any harmful impact on these structures would be expected to cause a reduction or thinning of the RNFL tissue. The contradictory results may be related to effects of age, or possible parallel effects on surrounding tissues (e.g., glial cells).

We find the side-specific correlation between the peripapillary RNFL and the rostral middle frontal gyrus volumes interesting; however, it is difficult to point out any clinical correlate to this finding.

To our knowledge, this is the first study to report OCT-findings in children prenatally exposed to OMT. Important strengths of this study are a high number of participants and a high rate of attendance. However, the use of different OCT instruments at the two study centres represents a challenge and a limitation. The large overrepresentation of smoking mothers in the OMT-group compared to the control group makes also a causal relation between the prenatal OMT-exposure and the OCT data uncertain.

To conclude, highly significant RNFL thinning was found in the prenatally OMT-exposed children at both study centres compared to the control groups. Macular thickness was also reduced, but not as unambiguously and explicitly as for the RNFL data. Our OCT-findings strongly suggest the impact on neurodevelopment from the prenatal OMT-exposure, although maternal smoking as a possible confounder cannot be ruled out. Further research on OCT-findings in prenatally OMT-exposed children is warranted.

## Supplementary Information

Below is the link to the electronic supplementary material.

Supplementary material 1 (DOCX 28.5 kb)

Supplementary material 2 (DOCX 29.1 kb)

## Data Availability

The data that support the findings of this study are available from the corresponding author upon reasonable request.

## Abbreviations

AMD: Age-related macular degeneration
ETDRS: Early treatment diabetic retinopathy study
FASD: Foetal alcohol spectrum disorder
OCT: Optical coherence tomography
OMD: Opioid maintenance therapy
RNFL: Retinal nerve fibre layer
