# Supplementary material for: Ocular fundus and optical coherence tomography (OCT) findings in children prenatally exposed to opioid maintenance therapy (OMT)
Source: BMC Ophthalmol. 2025 Nov 13;25:641. doi: 10.1186/s12886-025-04342-y (PMC12613939; doi:10.1186/s12886-025-04342-y)
Supplement: Supplementary file 1 — Supplementary material 1 (DOCX 28.5 kb) [file 12886_2025_4342_MOESM1_ESM.docx]

Supplement Table 1. Mean optic disc diameter (vertical and horizontal) in the OMT-exposed and the control group; data for centre A and B presented separately.

| Optic disc diameter (mm),  mean ± SD, eye and meridian | OMT-exposed group | Control group | p-value |
| --- | --- | --- | --- |
| Centre A | | | |
| Right eye  Vertical  Horizontal  Left eye  Vertical  Horizontal | 1.78 ± 0.41  1.60 ± 0.36  1.83 ± 0.20  1.65 ± 0.17 | 1.71 ± 0.41  1.57 ± 0.36  1.70 ± 0.40  1.59 ± 0.37 | 0.54  0.75  0.12  0.38 |
| Centre B | | | |
| Right eye  Vertical  Horizontal  Left eye  Vertical  Horizontal | 1.95 ± 0.20  1.83 ± 0.25  1.95 ± 0.20  1.81 ± 0.24 | 2.00 ± 0.17  1.91 ± 0.19  1.98 ± 0.12  1.88 ± 0.17 | 0.62  0.41  0.71  0.48 |
