# Supplementary material for: Ocular fundus and optical coherence tomography (OCT) findings in children prenatally exposed to opioid maintenance therapy (OMT)
Source: BMC Ophthalmol. 2025 Nov 13;25:641. doi: 10.1186/s12886-025-04342-y (PMC12613939; doi:10.1186/s12886-025-04342-y)
Supplement: Supplementary file 2 — Supplementary material 2 (DOCX 29.1 kb) [file 12886_2025_4342_MOESM2_ESM.docx]

Supplemental table 2. Peripapillary retinal nerve fibre layer (RNFL) thickness on optical coherence tomography (OCT) in the OMT-exposed group; methadone vs buprenorphine

| Retinal nerve fiber layer thickness (μ),  mean ± SD, eye and optic nerve head sector | Methadone | Buprenorphine | p-value |
| --- | --- | --- | --- |
| Centre A n=14 n=18 | | | |
| Right eye  General  Temporal  Temporal superior  Nasal superior  Nasal  Nasal inferior  Temporal inferior  Left eye  General  Temporal  Temporal superior  Nasal superior  Nasal  Nasal inferior  Temporal inferior | 95.4 ± 14.6  76.4 ± 8.9  133.5 ± 20.6  101.4 ± 24.4  77.1 ± 15.4  114.4 ± 39.1  124.6 ± 33.5  92.8 ± 14.1  68.8 ± 8.5  126.2 ± 22.8  109.4 ± 18.8  77.2 ± 20.6  111.4 ± 30.5  127.0 ± 9.5 | 97.6 ± 11.6  70.7 ± 7.1  140.0 ± 21.8  112.0 ± 18.7  79.6 ± 17.8  105.6 ± 28.9  141.8 ± 17.0  93.7 ± 11.5  67.8 ± 5.1  131.8 ± 13.1  115.6 ± 24.9  75.3 ± 18.0  100.4 ± 35.5  131.3 ± 18.3 | 0.70  0.12  0.50  0.26  0.74  0.55  0.12  0.89  0.78  0.50  0.62  0.84  0.54  0.62 |
